# Nox2 Is Required for Macrophage Chemotaxis towards CSF-1

**DOI:** 10.1371/journal.pone.0054869

**Published:** 2013-02-01

**Authors:** Sanjay Chaubey, Gareth E. Jones, Ajay M. Shah, Alison C. Cave, Claire M. Wells

**Affiliations:** 1 Cardiovascular Division, The James Black Centre, King’s College London BHF Centre of Excellence, London, United Kingdom; 2 Randall Division of Cell and Molecular Biophysics, King’s College London, London, United Kingdom; 3 Division of Cancer Studies, King’s College London, London, United Kingdom; The University of Hong Kong, Hong Kong

## Abstract

Macrophage migration and infiltration is an important first step in many pathophysiological processes, in particular inflammatory diseases. Redox modulation of the migratory signalling processes has been reported in endothelial cells, vascular smooth muscle cells and fibroblasts. However the redox modulation of the migratory process in macrophages and in particular that from the NADPH oxidase-2 (Nox2) dependent ROS has not been established. To investigate the potential role of Nox2 in the migratory response of macrophages, bone marrow derived macrophages were obtained from WT and NOX2 knockout mice (Nox2KO) and subjected to CSF-1 stimulation. We report here that loss of Nox2 expression in BMM resulted in a significant reduction in the CSF-1 induced spreading response suggesting that Nox2 can modulate cytoskeletal events. Moreover, Nox2KO BMMs were deficient in cellular displacement in the presence of CSF-1. More significantly, when challenged with a gradient of CSF-1, Nox2KO BMMs showed a complete loss of chemotaxis accompanied by a reduction in cell migration speed and directional migration persistence. These results point to a specific role for Nox2KO downstream of CSF-1 during the BMM migratory response. Indeed, we have further found that Nox2KO BMMs display a significant reduction in the levels of ERK1/2 phosphorylation following stimulation with CSF-1.Thus Nox2 is important in BMM cellular motion to CSF-1 stimulation and necessary for their directed migration towards a CSF-1 gradient, highlighting Nox2 dependent signalling as a potential anti-inflammatory target.

## Introduction

Several isoforms of the gp91^phox^ catalytic subunit of NADPH oxidase have been described. These isoforms are now termed NOXs, and comprise Nox1–5, Duox1 and 2 with Nox2 being the new name for gp91^phox^ [1]. Superoxide-generating enzymes are a major sources of ROS and have been shown, by way of redox modulation of cellular signalling, to play important roles in disease pathophysiology, in particular inflammatory diseases [2], [3], [4].

The progression of atherosclerosis is an inflammatory process requiring cellular migration and infiltration. Indeed, it has been shown that within atherosclerotic plaques, in ApoE^−/−^ mice, macrophages were a prominent source of Nox2 [5]. Furthermore, the Nox2 expression was elevated before the appearance of lesions, consistent with a causal role for the enzyme in the early activation of critical pro-atherogenic pathways. Importantly, global deletion of Nox2 in the ApoE^−/−^ mice inhibited atherosclerotic lesion development in the aortic arch, thoracic and abdominal aorta [5].

In keeping with atherosclerosis, a high cholesterol diet which is implicated in this process, has been shown to induce an inflammatory response in the post capillary venules [6]. This hypercholesterolemia induced inflammatory response was demonstrated to be dependent on superoxide production, in particular that from NADPH oxidase. Thus NADPH oxidase superoxide production is a critical event that initiates the leukocyte endothelial cell adhesion in postcapillary venules in mice following a high cholesterol diet [6].

Interestingly there is growing evidence in the literature for a role of the Nox family proteins in modulating the processes involved in cellular migration. For example, Rac stimulates actin polymerisation by several mechanisms including NADPH oxidase mediated ROS production [7]. The dephosphorylation of the cytoskeletal regulator cofilin following PDGF stimulation has also been shown to be Nox1 dependent [8], [9]. During fibronectin/integrin mediated cell adhesion, ROS is dramatically increased by Rac-1 dependent activation of NADPH oxidase [10]. Recently Nox4 has also been shown to be a key player in the regulation of stress fibre formation and focal adhesion turnover in VSMC [11]. NADPH generated ROS has also been shown to be important in invadopodia formation facilitating the invasive behaviour of cancer cells [12].

In keeping with the regulatory role of Nox2 in cellular migration, Rac1- and Nox2-dependent NADPH oxidase have been shown to play an important role in endothelial cell migration, as seen during tissue repair in response to injury, angiogenesis, and wound healing [13], [14], [15]. Also oxidised LDL, which extensively accumulates in atherosclerotic plaques, can stimulate ROS production in macrophages through NADPH oxidase, which stimulates downstream expression of proinflammatory cytokines. [16]. These cytokines have been shown to stimulate smooth muscle cell migration important in the progression of atherosclerotic plaques. However the direct role of Nox2 in the migration of macrophages, important in pathophysiological processes such as atherosclerosis and inflammatory diseases, has not been well established. This paper investigates whether the Nox2-dependent NADPH oxidase modulates the migration of macrophages and in particular to a common tissue chemoattractant, CSF-1.

## Materials and Methods

### Reagents

All chemicals and DMEM were purchased from Sigma. CSF-1 was purchased from RandD systems, USA. Versene for cell detachment was purchased from Gibco. Phalloidin-FITC was purchased from Sigma. Antibodies to phospho and total ERK1/2 and Akt were purchased from Cell Signalling Technology.

### Animal Husbandry and Maintenance

All mice were maintained in a designated facility in accordance with the Code of Practice for the Housing and Care of Animals Used in Scientific Procedures. Mice were housed up to a maximum of 5 per cage and had free access to water and normal food chow. The mice were anaesthetised using Isoflurane (2–2.5% isoflurane/oxygen). Once deep anaesthesia had been reached the mice were terminally culled by cervical dislocation. All experimental procedures were carried out under the authority of a Home Office Personal Licence and Project Licence. All animal procedures were performed following in accordance with the Guidance on the Operation of the Animals (Scientific Procedures) Act,1986 (UK Home Office) and approved by the King’s College London Animal Care and Use Committee.

### Isolation and Culture of Mouse Primary Bone Marrow Derived Macrophages

The murine femoral bones were harvested after the mice were culled using terminal anaesthesia. All the surrounding tissue on the bone was removed and the bone pierced at both ends with a 21-gauge needle. The bone marrow was flushed out of the bone with macrophage starve medium (RPMI 1640 with L-glutamine, 1% Essential amino acids, 1% sodium pyruvate, 1% P+S, 10% FCS and 0.5% βmercaptoethanol). Cells were then centrifuged and the pellet resuspended in macrophage starve medium. The cells were then counted and 2×10^5^ cells/cm^2^ seeded onto 10 cm petri dishes for 3 days in macrophage growth media (starve medium plus M-CSF1 at 30 ng/ml). After 3 days the non-adherent population of cells containing the monocytes was removed. The cells were centrifuged, resuspended and counted. The cells were then seeded onto 6-cm bacterial culture plates at a density of 10^5^ cells/mL. The cells are incubated for a further 5 days in the presence of M-CSF-1 as above. The differentiated BMMs become adherent and were harvested on day 5 for experimentation.

### Time-lapse Microscopy and Migration Analysis

To study random cell motion, cells were seeded onto 6 well plastic petri dishes at a density of 2×10^4^ cells/ml in macrophage growth medium and incubated overnight. Following incubation, cells were starved of CSF-1 in macrophage starve medium for 8 hours. The cells were then stimulated with CSF-1 by the re-introduction of CSF-1 (30 ng/ml) containing growth media. Cell images were collected using a Pulnix CCCD camera, taking a frame every 5 min for 18 h using AQM acquisition software (Andor, UK). Subsequently all the acquired time-lapse sequences were displayed as a movie and each cell in the first frame was tracked for the whole of the time-lapse sequence, using Motion Analysis software (Andor, UK) This resulted in the generation of a sequence of position co-ordinates relating to each cell in each frame. All the tracks were centred to co-ordinate (0,0) to better view the distance travelled. A circular horizon distance was set and the number of cells from the total population that reached the horizon distance was monitored. The random speed and the persistence in the random motion was calculated and compared. Mathematical analysis was carried out using Mathematica 6.0TM workbooks [17]. P-values less 0.05 were accepted as statistically significant.

To study chemotaxis, cells were seeded on acid washed coverslips at a density of 2×10^4^ cells/ml in macrophage growth medium and incubated overnight. Following incubation cells were starved of CSF-1 in macrophage starve medium for 8 hours. The coverslips were then mounted onto Dunn chemotaxis chambers as previously described [18] using recombinant murine CSF-1 (30 ng/ml) as the chemoattractant. Cell images were collected and analysed as described above.

### Immunofluoresence

BMMs were seeded on glass coverslips at 2×10^5^ cells per coverslip and maintained in macrophage growth or macrophage starve medium followed by CSF-1 stimulation as indicated. Cells were washed with PBS, fixed with 4% paraformaldehyde permeabilised and stained for actin using phalloidin-FITC. The actin images were collected on IX71 Olympus microscope and cell images were analysed using ImageJ.

### Immunoblotting

Cells were seeded onto 6 well plates and maintained or CSF-1 deprived as outlined above. Following stimulation cells were lysed as previously described [17] and lysates subjected to acrylamide gel electrophoresis as previously described [17]. Protein membranes were blocked and probed with primary and secondary antibodies as indicated. The blots were developed using Pierce® ECL Western Blotting Substrate Kit (Thermo Scientific). The autoradiograph were scanned and band densities were quantified with Kinetic Imaging software to obtain the ratio of phosphorylated protein to total protein.

## Results

### Nox2KO Macrophages have an Increased Spread Area

**Figure 1:**
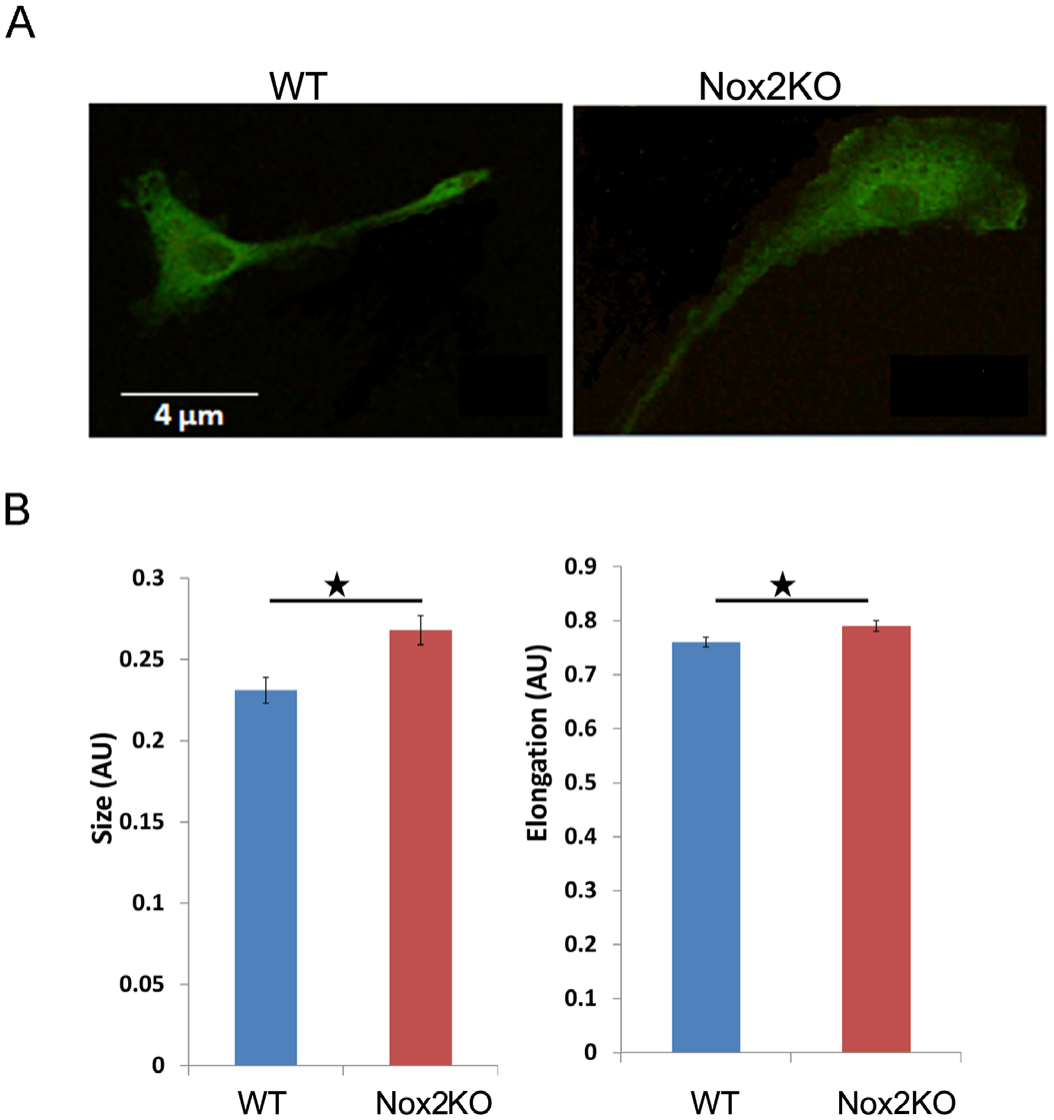
Nox2KO BMMs have increased cell area. a) WT and Nox2KO BMMs were fixed and stained for F-actin. b) Cell area and cell elongation analysis was conducted using ImageJ software. Representative of three independent experiments with >30 cells measured for each experiment.* = p<0.05.

**Figure 2:**
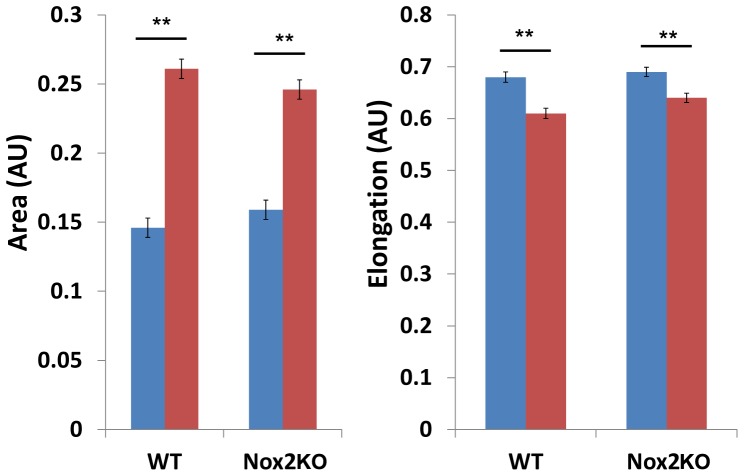
BMMs respond to CSF-1 stimulation. Cells were seeded on coverslips, CSF-1 deprived then re-stimulated with CSF-1 for 5 minutes. Cells were fixed and stained for F-actin and cell area and cell elongation analysis was conducted using ImageJ software. Representative of three independent experiments with >30 cells measured for each experiment. Where blue bars represent CSF-1 starved and red bars represent CSF-1 stimulated cells ** = p<0.005.

We were interested in establishing whether Nox2 plays a role in the infiltration of macrophages at sites of inflammatory response, such as those that are thought to be associated with tissue repair or conditions such as atherosclerosis. Many of the signalling pathways that regulate cellular migration are the same as those controlling cellular morphology. Therefore we first analysed the cell morphology of WT and macrophages derived from Nox2 knockout mice (Nox2KO) under basal growing conditions. We observed no difference in the global actin architecture between WT and Nox2KO BMMs but did find that Nox2KO BMM had a larger spread area and were reproducibly more elongated compared to WT BMM **(** **Figure 1a and b** **)**. CSF-1 is well known to stimulate cell morphology changes in BMMS [17], where deprivation of CSF-1 induces cell elongation and re-stimulation leads to centrifugal spreading [17]. Therefore we next tested whether Nox2 expression was required for a morphological response to CSF-1. Interestingly, following CSF-1 deprivation, WT and Nox2KO macrophages reduced their area to a similar size (Figure 2). Moreover, CSF-1 stimulation induced cell spreading in both WT and Nox2KO BMMs **(** **Figure 2** **)**. However whilst, WT macrophages exhibited an increase of 79% in spread area compared to their spread area under starved conditions Nox2KO macrophages only exhibited a 55% increase after 5 minutes of CSF-1 stimulation. Although not statistically significant, taken together with the reduced cell area for growing cells, these data do suggest that Nox2 expression is in part required for normal BMM behaviour. Interestingly, both populations were able to centrifugally spread in response to CSF-1 **(** **Figure 2** **)**, suggesting that CSF-1 responses are not completely dependent on Nox2 in BMMs.

### Nox2^−/−^ BMM Showed Reduced Random Motion Following CSF-1 Stimulation

**Figure 3.Nox2KO:**
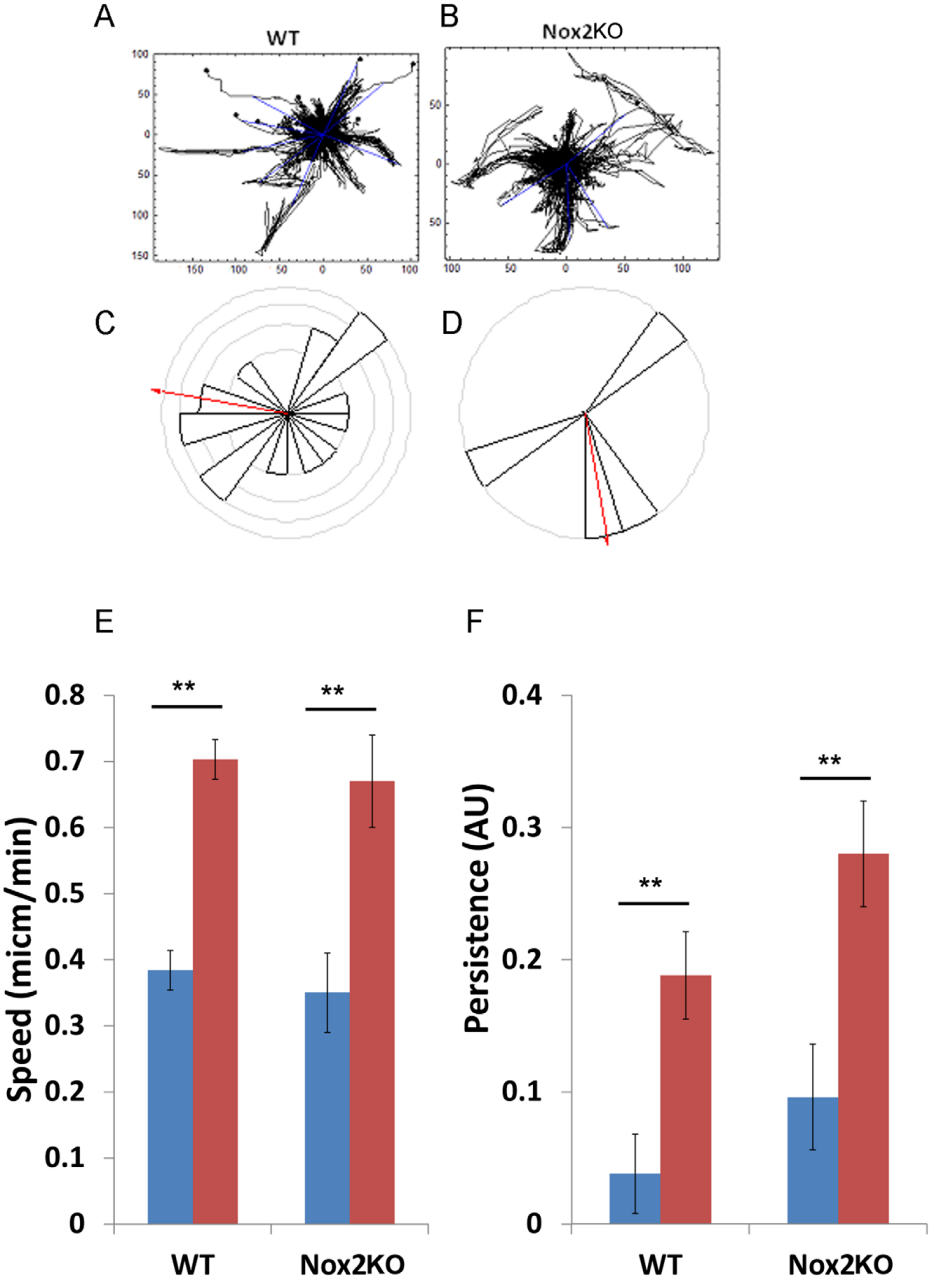
Nox2KO BMMs have reduced cell displacement. WT and Nox2KO BMMs were seeded on plastic, CSF-1 deprived then stimulated with CSF-1 and imaged as described in material and Methods. A and B) cell tracks of WT and Nox2KO BMM respectively. The tracks have been re-set to co-ordinate (0,0). C and D) The number of cells reaching a set circular horizon was monitored and found to be significantly (p = 0.02) more in the WT than Nox2KO BMM following CSF-1 stimulation. (E and F) mean cell migration speed and mean persistence of direction were calculated from cell tracks above (see material and methods for details). Where blue bars represent CSF-1 starved and red bars represent CSF-1 stimulated cells ** = p<0.005.

Given that we had observed a change in growing cell spread area and a reduction in the spread area in response to CSF-1, we speculated that Nox2KO BMMs might have defects in CSF-1 simulated migration. Initially we tested random migration and found that there was a small but reproducible reduction in cell migration speed observed in the Nox2KO population. In the presence of CSF-1 WT cells exhibited a mean migration speed of 0.71 µm/min whilst Nox2KO BMMs exhibited a mean migration speed of 0.67 µm/min **(** **Figure 3E** **).** Moreover we found a significant reduction (p = 0.02), in cell displacement **(** **Figure 3C and D** **)**. Where a reduced number of the Nox2KO BMMs population were able to reach the set horizon compared to WT BMMs. We speculate that this may be due in part to the slightly reduced cell speed but could also be attributed to the observation that Nox2KO BMMs tended to oscillate in movement more than WT BMMs and therefore not achieve overall displacement, this is partly reflected in the increased persistence of migration **(** **Figure 3F** **)** we recorded for the Nox2KO BMMs. These results suggest that Nox2 does play a role in the migration of BMMs following CSF-1 stimulation.

### Nox2^−/−^ Cells did not Exhibit a Chemotactic Response Towards CSF-1

**Figure 4:**
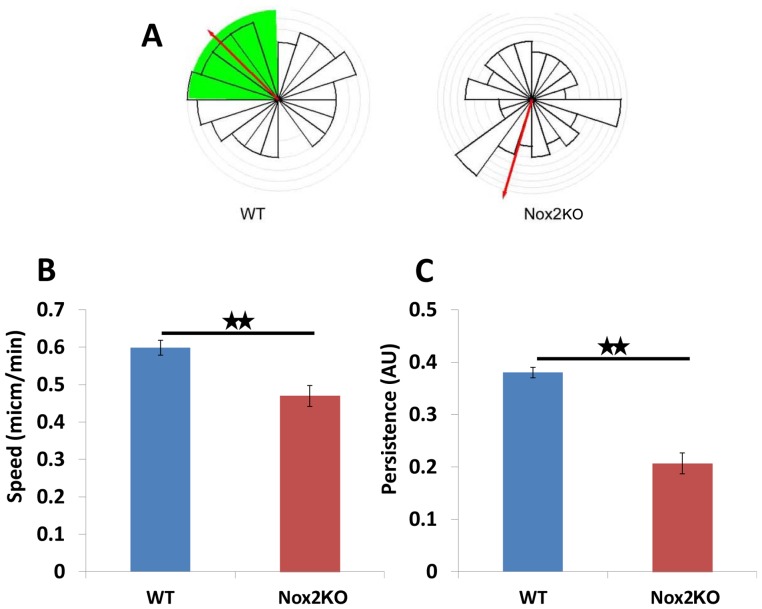
Nox2KO BMMs cannot chemotax towards a source of CSF-1. A) WT and Nox2KO BMMs were seeded on glass coverslips, deprived of CSF-1 and then placed in a gradient of CSF-1 using the Dunn chemotaxis chamber. Cells were tracked and the tracks re-set to co-ordinate (0,0) and represented by a circular histogram where the mean direction of cells is represented by a red arrow with 95% confidence interval (green wedge). Representative of three independent experiments. B and C) mean cell speed and mean persistence of direction were calculated from the tracks generated in (A). ** = p<0.001.

BMM are known to have a chemotactic response to CSF-1 [17], and in a physiological context are likely to be responding to a gradient of chemoattractant rather than global stimulation. Thus we next challenged the WT and Nox2KO BMMs to chemotax towards a source of CSF-1 using the Dunn Chemotaxis Chamber. Whilst WT BMM were able to efficiently chemotax towards CSF-1, Nox2KO BMMs completely lost their chemotactic response **(** **Figure 4** **)**. Loss of chemotaxis can sometimes be attributed to a reduction in cell speed and we did indeed find that there was a significant reduction in mean cell migration speed in the Nox2KO population (p<0.001) (**Figure 4C**). However, we found that cell persistence was also significantly (p<0.001) reduced in Nox2KO BMM **(** **Figure 4D** **)** as compared to WT suggesting that Nox2KO cells were unable to respond to the CSF-1 gradient. This would suggest a more significant role for Nox2 in the directed migration of the BMMs compared to random migration.

### Nox2^−/−^ Macrophages have an Attenuated Signalling Response to CSF-1

**Figure 5:**
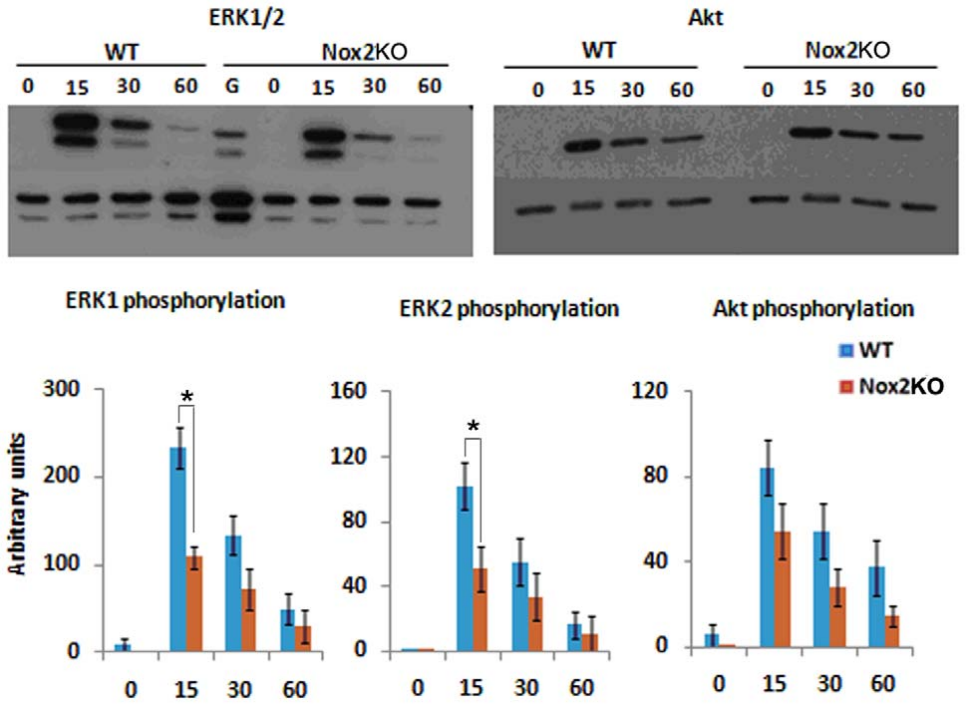
Nox2KO BMMs have reduced ERK phosphorylation downstream of CSF-1. A) WT and Nox2KO BMMs were CSF-1 deprived, then re-stimulated with CSF-1for the times indicated. Cells were lysed and probed for pAKt, pERK and total protein.B) autoradiographs were analysed using AndorIQ and levels of pERK1, pERK2 and pAKT were normalised to loading controls. Data represents three independent experiments. * = p<0.05.

Given that we have detected changes in both cellular morphology, cell spreading and directed cell migration we reasoned that signalling downstream of CSF-1 may be altered in Nox2KO cells. CSF-1 is well known to stimulate both ERK [19] and Akt phosphorylation [20] in BMMs. Indeed, levels of ERK phosphorylation have been linked to cell spreading [19]. We found no difference in Akt phosphorylation downstream of CSF-1, however, Nox2KO BMMs have an attenuated phospho-ERK response **(** **Figure 5** **)**. Where levels of ERK phosphorylation were significantly reduced following 15 mins of CSF-1 stimulation. Thus, Nox2KO BMM do have attenuated signalling downstream of CSF-1 stimulation.

## Discussion

The coordination and synergy between the cytoskeletal dynamics at the leading edge, the strengthening of adhesion to the ECM and cellular contractility play a key role in the dynamics of cellular morphology and migration [21]. Redox signalling has been shown to be influential in this process at many different stages. In this paper Nox2 has been shown to play a role in regulating cellular morphology, random cellular motion and also to be critical in directed cellular migration, speed and chemotaxis. A key finding in this paper was that Nox2 in BMM was found to be important in random cellular motion and necessary in directed cellular motion.

WT and Nox2KO BMMs are morphologically different and this is reflected in the difference in mean spread area of these cells and the elongated shape of Nox2KO BMMs. However, both populations were able to respond to CSF-1. Although, the Nox2KO BMMs tended towards a slower response in CSF-1 induced cell spreading. The loss of Nox2 did result in a significant reduction in the random motility of BMM as observed by the lower numbers of BMM migrating to the set horizon following CSF-1 stimulation. Also the Nox2KO BMM showed more intrinsic persistence in their random movement. Random motion allows cells to explore their environment. The increased intrinsic persistence suggested the loss of Nox2 had resulted in a reduction in the cells ability to turn and explore their environment. Pankov et al [22] demonstrated that total Rac1 activity was important in determining whether random cell migration followed a more intrinsic random or directionally persistent pattern of motion. The data here suggests that, at least in part, some of these regulatory functions of Rac1 could be through Nox2.

In contrast to random motion, directional migration moves cells rapidly between points. When challenged with a gradient, loss of Nox2 in BMM resulted in a complete loss of chemotaxis towards CSF-1 and a loss of cell migration and directional persistence. The BMM were able to sense and respond to CSF-1 stimulation as observed by the increase in the speed of WT and Nox2KO BMM, although Nox2KO BMMs were significantly slower than WT cells. Thus the loss of chemotaxis suggested a critical role for Nox2 further downstream from the cell sensing of the external signal and/or in cellular polarisation.

A possible mechanism by which Nox2 could affect the directionality of the cell could be by the redox modulation of the intracellular signalling gradients established by phophoinositides. The phosphoinositides PtdIns(3,4,5)P_3_ (PIP_3_) and PtdIns(3,4)P_2_[PI(3,4)P_2_] along with PI3K and PTENS are key signaling molecules in this process [23], [24]. This process involves both localized accumulation and activation of PI3Ks, which generate PIP_3_/PI(3,4)P_2_, and the phosphatase PTEN, which removes them [25]. Cells with altered PI3K or PTEN activity can usually show chemokinesis but exhibit a significantly reduced chemotaxis [24], [26]. Many of these signaling molecules have been shown to be redox sensitive. Leslie et al [27] demonstrated that oxidative stress with H_2_O_2_ resulted in the inactivation of PTEN. PTEN is a member of the Protein Tyrosine Phosphatase family which can be physiologically regulated through reversible oxidation resulting in their inactivation [28], [29]. The inactivation of PTEN results in an increase in cellular phosphoinositides and thus the loss of any gradient established by the phosphoinositides to a chemoattractant signal. Also phosphoinositides, PtdIns(3,4,5)P_3_ (PIP_3_) and PtdIns(3,4)P_2_[PI(3,4)P_2_], have been shown, by way of their Phox domains for subunits p40^phox^ and p47^phox^, to be involved in the recruitment and activation of these Nox regulatory proteins, [30], [31] thus establishing a means for the redox modulation of these downstream signalling molecules.

Cellular polarisation is equally important for directed cellular migration in which the small GTPase are important in this process and in particular Cdc42. Cdc42 is a master regulator of cell polarity by being active towards the front of migrating cells [32] and by restricting where lamellipodia forms [33]. Its importance is confirmed in experiments where both the inhibition and global activation of Cdc42 disrupts the directionality of migration [18], [34]. How Cdc42 and Nox2 are associated is not entirely clear however evidence from the literature suggest that in an *in-vitro* cell free experiment Cdc42 can act as a competitive inhibitor of Rac-1 and Rac-2 activation of cytochrome b_558_ and therefore ROS production [35]. Cell polarisation is reflected in the ability of a cell to modulate its shape during CSF-1 deprivation and re-stimulation.

It was interesting to note that whilst mean cell migration speed was significantly reduced during directed migration such a large difference in effect was not observed during random migration in the Nox2KO BMMs following global CSF-1 stimulation. The molecular mechanism for the Nox2 dependency on the speed of BMM migration is not established, but many of the proteins involved in the control of actin cytoskeleton reorganisation are redox sensitive such as PTENS and PI3K [36]. Lamellipodia formation in moving cells requires cycles of actin polymerization and depolymerisation. Rac stimulates actin polymerization by several mechanisms, including NADPH oxidase mediated ROS production [7].The relation between the actin cytoskeleton and ROS seems to be bi-directional. Thus, cortactin, an actin-binding protein that has traditionally been found to regulate polymerization of the actin cortex, has also been shown to mediate p47^phox^ translocation to the membrane during AngII induced activation of NADPH oxidase [37]. Moreover, actin activates Nox2 in neutrophils in a cell-free system, implying their direct effect on NADPH oxidase enzyme activity, and the destabilization of the actin cytoskeleton robustly enhances the neutrophil respiratory burst activity [38], [39]. A more complete understanding of this bidirectional relation between NADPH oxidases and the actin cytoskeleton may shed further light on how it mediates migration.

The significantly reduced phosphorylation of ERK1/2 was in line with its important role in cellular migration and that of Nox2 in the activation of Ras/Raf/MEK/ERK signalling cascade downstream from the tyrosine receptors. ERK1/2 localise to the cell membrane [40] and to focal adhesions [41] and promote lamellipodium formation and spreading in epithelial cells [42]. Smith et al found that ERK1/2 activity was reduced in PAK1^−/−^ BMMs which displayed spreading defects compared with WT BMMs thus suggesting that optimal activation of ERK1/2 is required during BMM spreading. [19] We also found reduced activation of ERK1/2 in the Nox2KO BMM following CSF-1 stimulation suggesting a possible mechanism whereby Nox2 generated ROS is able to modulate the downstream response via activation of ERK.

Our data points to an involvement of NOX2 in BMM migration. It is interesting to note that different isoforms of NADPH oxidase have also been shown to be involved in the migration of other cell types. Nox4 has also recently been found to be a key player in the regulation of stress fibre formation and focal adhesion turnover in VSMCs [11]. These findings suggest a potentially novel mechanism of local ROS production by which focal adhesion turnover is coordinated. Certainly a role of Nox2 in the regulation of such adhesion formation in BMM could explain the difference observed in their shape and then in their speed and persistence. Further studies of differences in the expression of integrins would increase the understanding of the exact underlying mechanism whereby the loss of Nox2 results in a reduction in the speed of migration in BMM. An important role for Nox1 in the migration of VSMC to βFGF agonist stimulation has also been identified [43] in rat SMC where inhibition of Nox1 significantly blocked migration.

In summary in order to initiate inflammation and tissue repair, the migration of macrophages into tissue is an important initial step. However the loss of Nox2 results in significant reduction in the random migration of BMM. On interrogating the BMM towards a directed target we have shown that the loss of Nox2 proved crucial as its loss resulted in the complete loss of chemotaxis. Nox2 was also important in the BMM speed and persistence towards a CSF-1 gradient with significant reductions in both. This loss of Nox2 also manifested itself in a reduced ERK1/2 phosphorylation and spreading responses to CSF-1 stimulation.

### Concluding Remarks

We have investigated for the first time the role of Nox2 in macrophage migration. Data presented here indicates Nox2 expression is necessary in response to CSF-1 stimulated migration. This *in-vitro* behaviour could in part be related to in vivo phenotypes associated with Nox2. A complete deficiency of Nox2, as in patients with chronic granulomatous disease (CGD), is associated with hyperinflammation, suggesting that the normal functions of Nox2 in macrophages and potentially other inflammatory cells are essential in restricting or resolving inflammation. On the other hand, Nox2KO mice are protected against fibrosis that accompanies inflammatory repair processes in the liver [44], [45], heart [46], [47], [48] and kidneys [49], [50]. Furthermore, specific inhibition of Nox2 reduces macrophage infiltration into vessels in a model of angiotensin II-induced hypertension [51] whilst macrophages lacking Nox2 oxidase activity are reported to infiltrate less efficiently into atherosclerotic lesions [52] and the aorta [53]. No mechanisms to explain these observations were reported in these studies. Our current results suggest that Nox2-dependent regulation of macrophage migration may underlie the effects on macrophage infiltration previously reported in experimental models of atherosclerosis and vascular disease. They further suggest that inhibition of Nox2 may be beneficial in such settings (all vascular disease) by inhibiting inflammatory infiltration. The development of novel therapeutics will however require a clear understanding of how this relationship is regulated.
